# Thermophilic microbiome acclimation for enhanced anaerobic digestion of food waste: Optimization and performance evaluation

**DOI:** 10.1371/journal.pone.0336355

**Published:** 2025-11-10

**Authors:** Hengxuan Shao, Chunle Yuan, Jingwen Qiang, Wei Hua, Yanling Cheng, Wanqing Wang

**Affiliations:** 1 Biochemical Engineering College, Beijing Union University, Beijing, China; 2 Beijing Key Laboratory of Biomass Waste Resource Utilization, Beijing, China; Tsinghua University, CHINA

## Abstract

Thermophilic anaerobic digestion (TAD) represents a promising approach for food waste (FW) treatment, offering significant advantages including accelerated reaction rates and increased volumetric biogas yield. However, the practical application of TAD is hindered by both the limited availability of thermophilic methanogenic consortia and heightened sensitivity to organic loading rate (OLR) fluctuations. In this study, a two-stage temperature shift strategy from mesophilic to thermophilic was implemented to establish a stable methanogenic community. The results showed that daily biogas yield increased steadily with rising fermentation temperature, reaching to a peak of 671.2 ml at 55 °C, which were 60.8% higher than that of mesophilic digestion. Microbial community analysis revealed that TAD increased the abundance of dominant hydrolytic bacteria (*e.g., Defluviitoga*) and hydrogenotrophic methanogen (*e.g., Methanoculleus*), consequently enhancing biogas production efficiency. Moreover, gradually increasing the OLR from 1.5 to 4 g VS/(L·d) significantly enhanced both biogas production and CH_4_ content, achieving a peak daily biogas yield of 2264.8 ml with sustained CH_4_ concentration stability (72–76%).

## Introduction

Food waste (FW), a major component of municipal solid waste, includes food processing waste and edible residues mainly produced by families, canteens and catering industries [1]. FW is characterized by high organic content and high moisture which is an extremely promising substrate for the biogas generation process. The high moisture content (76.3%–92.4%) endows it with readily biodegradable characteristics, while the high organic content (about 90% VS, volatile solids) makes it as a valuable medium for bacteria [2]. Anaerobic digestion (AD) is an effective way for FW disposal, enabling the full quantitative utilization of FW. During the processing, FW is converted into three products: biogas for renewable energy, solid digestate as an organic fertilizer, and liquid digestate. Subsequently, this liquid fraction can be treated using a low-cost, low-energy algal-bacterial symbiotic system, which facilitates the co-production of valuable algal biomass alongside nutrient removal [3]. This integrated strategy valorizes all outputs, achieving maximum resource recovery and minimal waste.

AD represents a promising technology for organic waste valorization and renewable energy production; however, its widespread adoption is often hampered by challenges related to process instability and inefficient biogas generation [4–5]. The process’s efficiency and stability are critically dependent on a delicate interplay of key operational parameters. These include maintaining a stable pH (typically 6.5–8.0) to prevent the inhibitory accumulation of volatile fatty acids (VFAs), ensuring a balanced carbon-to-nitrogen (C/N) ratio (ideally 20–30:1) to avert ammonia toxicity or nutrient deficiency, and meticulously managing the OLR and hydraulic retention time (HRT) to avoid system overload and ensure complete substrate degradation [2,6]. Among these variables, temperature emerges as a critical operational parameter, exerting direct control over the metabolic rates of microbial consortia, digestate viscosity, and the overall kinetics of biogas production. Elevated temperatures enhance the solubilization of organic matter, improve substrate conversion efficiency, and accelerate subsequent methanogenesis by modulating microbial kinetics and thermodynamic equilibria [7]. Consequently, AD is typically conducted under either mesophilic (MAD, 35–37 °C) or thermophilic (TAD, 55–60 °C) conditions [8]. Relative to its mesophilic counterpart, TAD offers distinct advantages, including accelerated hydrolysis rates, which permit shorter retention times and higher biogas yields, alongside more effective pathogen inactivation in the final digestate [9].

Despite its recognized advantages, TAD is principally constrained by its heightened sensitivity to organic overloading and the inherent thermosensitivity of methanogenic archaea. Elevated OLR in TAD systems can readily induce the accumulation of VFAs, culminating in process instability and failure. It was found that TAD outperformed MAD as the OLR increased from 2.5 to 5.5 g VS/(L·d), but process inhibition occurred at an excessive OLR of 6.5 g VS/(L·d) [10]. Moreover, the thermophilic hydrolysis phase exhibited limited buffering capacity, rendering the system vulnerable to both acid shock and thermal stress [11]. In thermophilic dry anaerobic digestion of FW, the maximum OLR tolerance was limited to 4 g VS/(L·d) due to ammonia nitrogen inhibition and VFA accumulation [12]. Therefore, systematic investigation of FW thermophilic anaerobic digestion characteristics under varying OLRs is critical for enhancing system stability. A second major operational challenge of TAD systems stems from the scarcity of thermally acclimated inoculum, which prolongs startup periods and elevates the risk of process failure [13]. Previous studies have demonstrated that methanogens are often severely impacted by temperature shock, which subsequently leads to kinetic uncoupling within the biological steps [14]*.* To establish stable and efficient thermophilic digester microbiomes, mesophilic anaerobic sludge is widely used as the predominant inoculum due to its rich microbial diversity, wherein a subset of facultative thermophiles can rapidly adapt to elevated temperatures [13]. Commonly, two temperature transition strategies are adopted to switch from mesophilic to thermophilic systems: a sharp shift (one-step) and a mild shift (stepwise) [15–16]. Previous studies have shown that, a one-step temperature shift selectively enriched thermophilic bacteria and mesophilic archaea, the stepwise transition strategy fostered a more balanced microbial consortium, ultimately resulting in enhanced methane (CH_4_) production [13].

In light of the limited availability of mature inocula from industrial-scale thermophilic digesters, this study selected mesophilic anaerobic sludge from the well-established Dongcun Integrated Treatment Plant. To adapt this inoculum, we employed a two-stage temperature control strategy to cultivate a high-efficiency thermophilic consortium for food waste anaerobic digestion. The initial stage involved a one-step temperature increase to 50 °C, leveraging strong selective pressure to preferentially enrich thermophilic hydrolytic-acidifying bacteria for efficient substrate degradation while preserving temperature-tolerant mesophilic methanogens. Subsequently, to fine-tune the microbial community and ensure long-term stability, the strategy transitioned to a prudent, stepwise temperature increase. This phase was critical for protecting and acclimating environmentally sensitive methanogens and maintaining the dynamic balance between acidogenesis and methanogenesis. These measures were key to establishing a structurally balanced, operationally controllable thermophilic digestion system, ultimately achieving a significant enhancement in methane yield. To elucidate the mechanisms underlying this performance, we analyzed the microbial community composition throughout the stepwise temperature elevation. Furthermore, the system’s tolerance to increasing OLRs was systematically investigated. The correlations between methane yield and critical process parameters (e.g., pH, VFAs) were comprehensively analyzed during the phased OLR increase, with the ultimate goal of developing optimization strategies to enhance the stability and long-term operational viability of TAD systems.

## Materials and methods

### Substrates and inoculum

The fermentation substrate, hereafter referred to as food waste (FW), was formulated to mimic the primary components of actual kitchen waste (KW) collected from residential Food Waste Bins in the Fatou Community of Beijing, China. The collected KW typically comprised uncooked vegetables, fruit peels, cooked rice, noodles, and meat. The formulated FW consisted of vegetables, distilled water, cooked rice, potato peels, and cooked chicken in a weight ratio of 26:10:10:4:1.

To ensure stable and efficient anaerobic digestion, the C/N ratio, a critical process parameter, was adjusted to an optimal 20:1 by augmenting the proportion of potato peels. Following formulation, the ingredients were mixed, chopped, and homogenized using an electric blender to a particle size of <5 mm. The resulting slurry, along with similarly homogenized KW samples, was stored at 4°C until use. The final substrate was characterized by a Total Solids (TS) content of 17.7% (wet weight basis) and a Volatile Solids (VS) content representing 94.7% of the TS.

The inoculum was obtained from the Dongcun Integrated Waste Treatment Plant in China. Prior to the experiment, the sludge was incubated anaerobically at ambient temperature for two weeks to deplete residual biodegradable matter and minimize endogenous methane production. The detailed physicochemical characteristics of the prepared substrate and the inoculum are summarized in Table 1.

**Table 1:** Characteristics of residential kitchen waste, food waste and inoculum.

Parameters	Kitchen waste	Food waste	Inoculum
TS (%, wet basis)	23.3 ± 0.39	17.7 ± 0.64	3.2 ± 0.25
VS (%, wet basis)	22.1 ± 0.45	16.7 ± 0.12	1.32 ± 0.12
VS/TS (%)	94.8 ± 0.42	94.7 ± 0.22	41.2 ± 0.21
TOC (%, dry basis)	52.3 ± 2.17	41.5 ± 3.06	25.8 ± 0.2
TN (%, dry basis)	4.1 ± 0.11	2.0 ± 0.14	3.5 ± 0.2
C/N	12.7 ± 0.02	20.6 ± 0.23	7.4 ± 0.3
pH	5.36 ± 0.05	4.25 ± 0.02	8.37 ± 0.01

TS: total solids, VS: volatile solids, TOC: total organic carbon, TN: total nitrogen.

### Batch thermophilic anaerobic digestion

**Fig 1:**
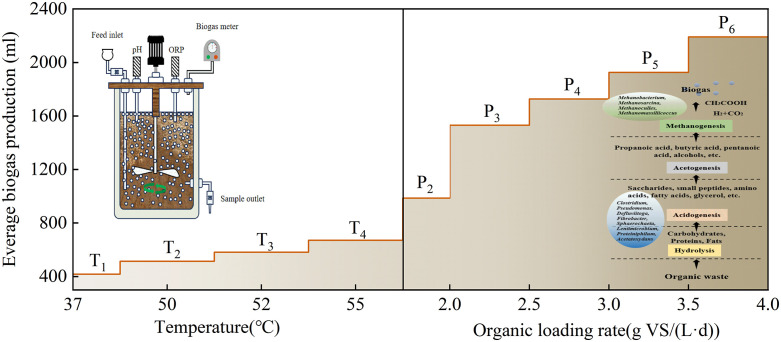
Schematic diagram of the thermophilic anaerobic reactor and experimental process design.

A schematic representation of thermophilic anaerobic digestion system is shown in Fig 1.

Batch experiments were performed in a 1 L custom-fabricated stirred-tank reactor (Wuhan Ruyi Organic Facility, Wuhan, China) with a 0.8 L working volume. Anaerobic conditions were ensured by nitrogen purging for 20 min before starting the experiment. Then an air bath shaker (80 rpm) was used for batch fermentation. For the thermophilic anaerobic fermentation, the acclimatization temperature was gradually increased from 37 °C to 60 °C and the substrate was fed every day at OLR of 1.5 g VS/(L·d) to enrich the thermophilic anaerobic microorganisms. Finally, the optimal anaerobic digestion temperature of 55 °C was chosen through both microbial community diversity and methane production analysis. To ensure stable operation, the starting OLR for the TAD systems was set at 1.5 g VS/(L·d), and then gradually increased to 5.0 g VS/(L·d). The whole experiment was divided into three typical periods (start-up phase, steady-state phase and collapse phase), and each period represented a different OLR (1.5, 2–3.5 and 4–5 g VS/(L·d)). During the digestion process, biogas production, methane content, pH and volatile fatty acids (VFAs) were monitored daily. All experiments were carried out in triplicate.

The organic loading rate was calculated by Eq. (1)


$$OLR=\frac{Mfeed\times \%TS\times \%VS}{Vdigester}$$ (1)

OLR: organic loading rate (units: kg VS/m³/day); M_feed: mass of kitchen waste fed per day (units: kg/day); %TS: percentage of total solids in the feed waste; %VS: percentage of volatile solids within the total solids; V_digester: working volume of the digester (units: m³).

### Analytical methods

Total solids (TS) and volatile solids (VS) contents were determined gravimetrically according to Standard Methods. The elemental composition of the substrate, specifically total organic carbon (TOC) and total nitrogen (TN), was analyzed using an elemental analyzer (Vario MACRO cube, Elementar Analysensysteme GmbH, Langenselbold, Germany).. The organic matter removal and conversion rates were calculated according to the method published by Zhang [8].

Biogas was collected in biogas sampling bags (Labshark, Hunan), and the volume was measured by a biogas flowmeter. The volumes of biogas were calibrated to volumes at standard conditions (273 K, 1 atm). The biogas composition (CH_4_ and CO_2_) was analyzed with a gas chromatograph (GC) (2014, Shimadzu, Japan) equipped with a thermal conductivity detector (TCD) and a TDX-01 stainless steel packed column (3.0 m × 3.0 mm, Shimadzu, Japan). The temperatures of the injector, column oven, and detector were set to 200°C, 100°C and 200°C, respectively. The pH of the samples was measured with a pH meter (PB-10, Sartorius) at room temperature. The volatile fatty acids (VFAs, including acetic acid, propanoic acid, butanoic acid, and pentanoic acid) were analyzed by HPLC (LC-20A, Shimadzu, Japan) equipped with an ion exchange column (Aminex HPX-87H, BioRad) and a refractive index detector (RID-20A). A mobile phase of 15 mM H_2_SO_4_ at a 0.5 ml min^-1^ flow rate was used.

### Microbial community and data analysis

Frist, substrate and digestate across different fermentation temperature conditions (37 °C, 50 °C, 52 °C, 55 °C and 60 °C) were sampled to characterize the adaptation process of thermophilic microbiota. Then, digestate samples from steady-state phase of TAD (55 °C, OLR: 3.5 g VS/(L·d)) were collected to analyzing the steady-state microbial community characteristics. The DNA extraction, construction of 16S amplicon libraries, and Illumina Novaseq sequencing followed established protocols [17]. The hypervariable V3-V4 region of the 16S rRNA gene was amplified with the primer pair 515F and 806R. 16S rRNA gene sequencing was conducted on an Illumina PE300 (Anoroad, Co., Ltd, Beijing, China). Quality control, splicing and clustering of raw sequenced sequences were performed to obtain OTUs, followed by species taxonomic annotation. The raw sequences have been deposited in the NCBI Sequence Read Archive under accession number PRJNA1310761.

### Statistical analysis

All experiments were performed in triplicate, and the results are presented as the mean ± standard deviation (SD). Statistical analyses, including the homogeneity of variance test (Levene’s test) and Student’s t-tests, were conducted using SPSS software (Version 24.0, IBM Corp, Armonk, NY, USA). A p-value of < 0.05 was considered to indicate a statistically significant difference. Redundancy analysis (RDA) was performed to explore the relationships between microbial community structure and environmental variables using Canoco 5 software. All figures were generated using Originpro 2024 (OriginLab Corporation, Northampton, MA, USA).

## Results

### Influence of temperature on biogas production and methane content

The physicochemical properties of the substrates are detailed in Table 1. Both the collected KW and the formulated FW were characterized by a high organic content, as evidenced by their VS/TS ratios exceeding 94%. However, the raw KW presented a critical limitation for stable digestion: a low C/N ratio of 12.7, which poses a significant risk of process inhibition due to ammonia accumulation. To overcome this limitation, the FW was specifically formulated by augmenting the proportion of potato peels, thereby achieving an optimized C/N ratio of 20.6. This adjusted value falls squarely within the optimal range of 20–30 for stable methanogenesis [18], thus confirming the suitability of the prepared FW as a representative yet nutritionally balanced substrate for this study.

**Fig 2:**
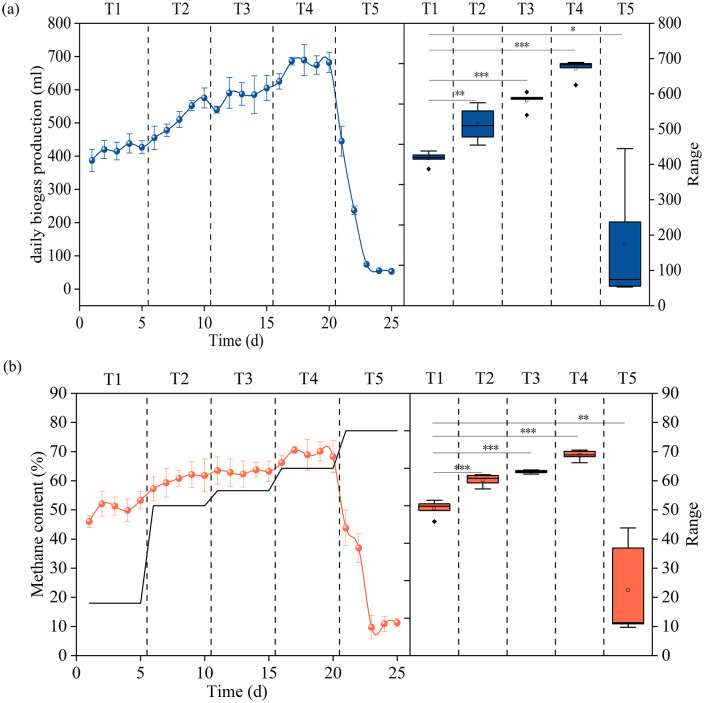
Performance of daily biogas production and methane content under different temperatures. (a) Daily biogas yields and average biogas yields under different temperatures; (b) CH_4_ volume concentrations and average CH_4_ concentrations under different temperatures.

A high-performance thermophilic consortium was successfully enriched in continuously stirred-tank reactors (CSTRs) over a 25-day acclimation period via a two-stage temperature shift strategy. The stability and efficiency of the digestion process were continuously monitored by measuring the daily biogas yield and methane concentration. Throughout the temperature ramp-up from mesophilic (37 °C) to thermophilic (55 °C) conditions, the daily biogas production exhibited a generally increasing, albeit fluctuating, trend across stages T1 to T4 (Fig 2a). Significant process variability was observed, particularly during the initial abrupt temperature shift from 37 °C to 50 °C, suggesting a period of microbial acclimation and enhanced thermotolerance development. The system’s performance culminated on day 20 at the T4 stage, reaching a peak daily biogas production of 671.2 mL. This represented a substantial 60.8% improvement over the initial performance at the T1 stage (417.4 mL), suggesting the establishment of a well-acclimated and highly efficient thermophilic microbial community. However, a further temperature increase to 60°C proved to be inhibitory, leading to a sharp decline in biogas production (<60 mL) and subsequent failure of the TAD process.

The methane content in biogas served as another significant indicator for assessing both biogas quality and methanogens activity. As Fig 2b shown, the methane content increased steadily with rising fermentation temperature, reached to a peak at 55 °C. The average CH_4_ concentration increased significantly from 50.5% to 68.8% during the temperature transition from 37 °C to 55 °C. These results agree with the documented CH_4_ concentration range of 50%−70% in similar systems [19]. Nevertheless, upon continued heating to 60 °C, the CH_4_ content declined to 22.5% along with a reduction in biogas production suggesting that 60 °C exceeded the thermal tolerance threshold of most bacteria and archaea, ultimately causing microbial community dysbiosis in the system. Other study also shown that temperature-phased anaerobic digestion (TPAD) with a first phase AD at 55 °C followed obtained a higher CH_4_ generation [20]. Similarly, reported that pre-treatment at 55 °C for 2 days followed by AD at 55 °C produced 106% more biogas than the 35–35 °C condition [21].

During anaerobic digestion, substrate organic matter is biochemically converted to VFAs and other intermediates, which are subsequently converted to biogas by methanogens. The organic matter removal rate reflects the efficiency of hydrolytic breakdown, while the organic matter conversion rate indicates the effectiveness of biogas production [22]. In this study, both rates were determined based on volatile solids (VS) content to assess overall microbial metabolic activity and process efficiency, with the results presented in S2 Fig. During the transition from mesophilic to thermophilic conditions, the organic matter removal rate increased markedly from 40.3% at 37 °C to 58.5%, and subsequently peaked at 76.2% at 55 °C. This enhancement is consistent with both earlier and more recent findings that elevated temperatures enhance hydrolysis rates and stimulate the activity of hydrolytic and acidogenic bacteria, thereby leading to higher solids degradation efficiencies [22–23]. In contrast, the organic matter conversion rate showed only minor changes, with a slight decline during the early thermophilic stage, suggesting that methanogenic archaeal activity was temporarily inhibited by the sudden temperature upshift—likely due to their narrower optimal growth temperature range and lower thermal tolerance [24]. With continued operation, methanogens gradually adapted to the thermophilic environment and reached a stable methanogenesis phase [22]. However, when the temperature was abruptly raised to 60 °C, both the removal and conversion rates declined markedly, suggesting that excessive thermal stress suppressed overall microbial growth and metabolic activity, consistent with previous observations.

Beyond the steady-state performance, the dynamics of the thermal ramp-up process itself provided crucial insights into the community’s adaptive capacity. A brief but discernible lag phase of approximately 2–3 days was observed during the initial transition from mesophilic (37 °C) to thermophilic (50 °C) conditions. This was evidenced by a temporary dip in biogas production and a slight disruption in methane content before both parameters recovered. In contrast, once a robust thermophilic community was established, subsequent stepwise temperature increases from 50 °C to 55 °C were seamless, with no discernible lag phase. This demonstrates that the established thermophilic community was highly resilient and adapted immediately to incremental temperature changes within its optimal range.

In summary, these findings demonstrate that while temperature elevation can effectively enhance digestion performance, an optimal threshold exists. Our results identify 55 °C as the optimal fermentation temperature that accelerates hydrolysis while maintaining microbial stability. These insights have direct implications for developing operational ramp-up strategies. Practitioners should anticipate an initial acclimation period of approximately 3 days when first transitioning to thermophilic conditions. Subsequently, gradual temperature increases within the 50–55 °C range can be implemented with minimal to no lag time, enabling rapid and efficient process optimization.

### Dynamic profiles of pH and VFAs concentration during the thermal ramp-up phase

**Fig 3:**
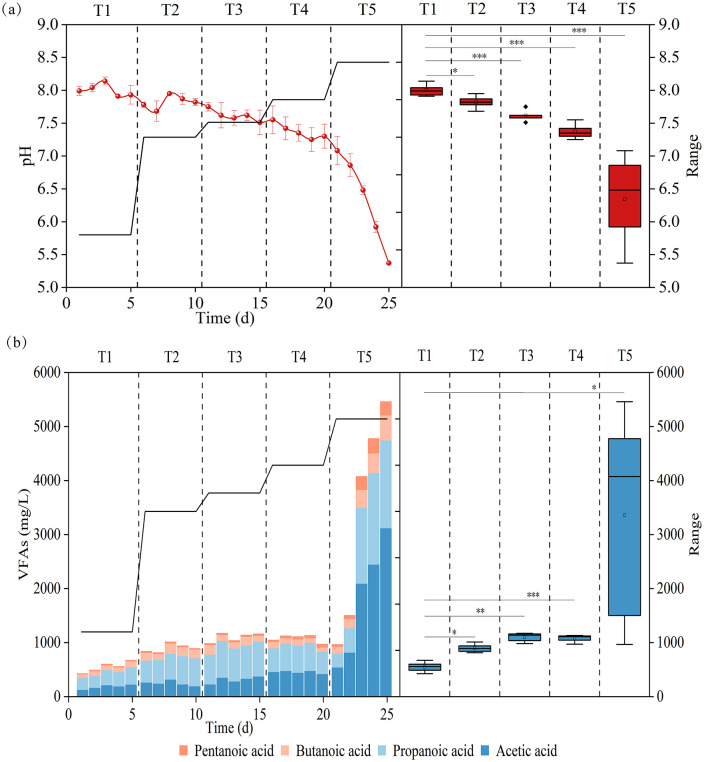
Changes in pH and VFAs throughout thermal transition. (a) Changes in pH and average pH under different temperatures; (b) VFA concentrations and average TVFA concentrations under different temperatures.

The pH value is a key parameter for ensuring fermenter stability. In the AD process, the accumulation of VFAs causes pH fluctuations, impairing microbial activity and causing system acidification, thereby reducing biogas production and compromising process stability [25]. Hence, closely monitoring pH and VFA concentrations is crucial for maintaining anaerobic digestion system stability. As the temperature increased, pH exhibited an overall descending trend (Fig 3a). The average pH value in 55 °C thermophilic AD (7.37) was slightly lower than that in 37 °C mesophilic AD (8.00), with both values remaining within the optimal range for biogas fermentation. Previous studies indicated that digesting microorganisms remain metabolically active within a pH range of 5.5–8.5, with optimal metabolic activity occurring at pH 6.5–8.0 [26]. However, when temperature was elevated to 60 °C, pH declined rapidly, reaching 5.3 on day 3rd of 60 °C fermentation, indicating probable process acidification.

The change in pH is due to changes in VFAs concentration. During the temperature ramp-up to 55 °C, VFA concentrations showed a slight increasing trend, consistent with pH changes, while maintaining stable low levels (<1000 mg/L) across all temperature phases (Fig 3b). Acetic acid and propionic acid emerged as the predominant digestion products throughout the mesophilic-to-thermophilic transition. The average concentrations of acetic acid and propionic acid in thermophilic AD (55 °C) were 2.4-fold and 1.8-fold higher, respectively, than those in mesophilic AD (37 °C), whereas butyric acid and valeric acid were barely detectable. This distinct VFA profile, characterized by the predominant production of acetic and propionic acid over longer-chain fatty acids, stems from thermally accelerated hydrolytic processes. The selective generation of these short-chain volatile fatty acids provides ideal substrates for methanogenic archaea, thereby enabling efficient biogas production and long-term operational stability. Consistent with Ahring’s findings, the concentrations of butyrate and isobutyrate serve as reliable indicators of system instability under thermophilic conditions [27]. However, when the temperature was elevated to 60 °C, the total VFA concentration increased sharply to 5461.86 mg/L, causing a precipitous pH decline and subsequent process collapse. Previous studies have demonstrated that VFA concentrations exceeding 4 g/L in anaerobic bioreactors lead to biogas production inhibition [28]. Similarly, the elevated VFA concentration in the 60 °C digestion system exceeded the tolerance limit of methanogens, disrupting the acidogenic-methanogenic balance and ultimately reducing biogas yield.Collectively, temperature significantly influenced pH dynamics. Operating at 55 °C significantly boosted both hydrolytic and methanogenic activity. And it optimized AD performance through increased acetic acid production and rapid pH stabilization.

### Influence of temperature on microbial community composition

To investigate the effect of temperature on microbial community development during the thermal ramp-up process, 16S rRNA gene sequencing was used to identify key microbial communities in the bioreactors. Samples from days 0, 5, 10,15, 20 and 25 corresponded to T0 (control: inoculum), T1, T2, T3, T4 and T5, respectively. Alpha diversity analysis revealed a clear, temperature-dependent trend. Community richness (Chao1), diversity (Shannon), and evenness (Simpson) all increased progressively, peaking at 52 °C (T3) before declining at higher temperatures (S1 Appendix). This peak in alpha diversity, which is often indicative of a stable and efficient anaerobic digestion system, coincided with the enhanced performance of our thermophilic process. Beyond these changes in complexity, beta diversity analysis revealed a fundamental reorganization of the community structure. A Principal Component Analysis (PCA) showed that temperature was the primary driver of this variation, starkly segregating the communities into a low-temperature group (inoculum, 37 °C, 50 °C) and a high-temperature group (52 °C, 55 °C, 60 °C) (S1 Fig). This profound structural divergence was statistically significant (PERMANOVA, p < 0.001) and primarily driven by the first principal component (PC1, 67.05% of variance). Together, these results highlight a critical ecological threshold between 50 °C and 52 °C. Above this point, intense heat-induced selective pressure appears to filter out the original mesophilic consortia, fostering the dominance of a distinct and highly stable thermophilic community.

**Fig 4:**
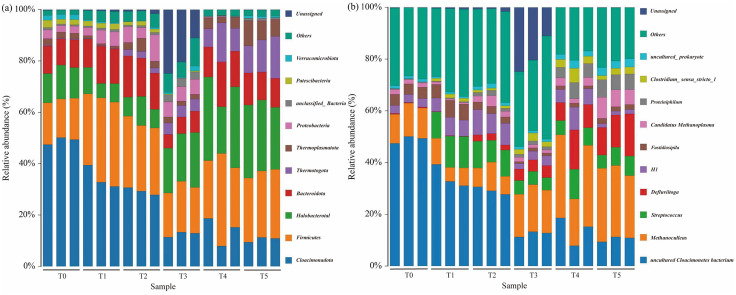
Changes in the relative abundance of the microbial community at the (a) phylum and (b) genus levels throughout thermal transition.

Analysis of the thermophilic reactor microbiome revealed a core bacterial community predominantly composed of four key phyla: *Firmicutes*, *Thermotogota*, *Cloacimonadota*, and *Halobacterota* (Fig 4a). These phyla are collectively responsible for the critical upstream processes of hydrolysis and acidogenesis, as well as essential syntrophic interactions that facilitate methane production. The phylum *Firmicutes* was identified as a dominant member of the thermophilic community, a finding consistent with its established role in various anaerobic digestion systems. The primary function of *Firmicutes* is the initial degradation of complex organic substrates and the subsequent production of VFAs. This is achieved through the secretion of a diverse array of extracellular enzymes that hydrolyze biopolymers into simpler, fermentable monomers [29]. Characterized by their thick peptidoglycan cell walls, their prevalence underscores their robust adaptation to the thermophilic anaerobic environment. The phylum *Thermotogota* plays a crucial role in maintaining system stability, particularly through its involvement in cellulose degradation [30–31]. The significance of this phylum was further demonstrated by its substantial increase in relative abundance from 0.17% at 37°C to 12.9% at 60 °C during the thermal ramp-up process, indicating its strong positive selection under increasing thermophilic conditions. This adaptation is critical for the efficient breakdown of recalcitrant Lignocellulosic materials often found in food waste. Members of the phylum *Cloacimonadota* were identified as putative syntrophs, engaging in essential metabolic interactions with methanogenic archaea. This syntrophic relationship is critical for the degradation of intermediate fatty acids, a key rate-limiting step in anaerobic digestion. By efficiently oxidizing these intermediates, *Cloacimonadota* facilitates a thermodynamically favorable environment for methanogens, thereby contributing directly to the optimization of subsequent methane yields [32–33]. The phylum *Halobacterota*, typically adapted to hypersaline environments, was found to contribute to methanogenesis via the methylotrophic pathway. This function has been observed in representative genera such as *Methanohalobium*, highlighting an alternative route for methane production within the community that complements the more dominant hydrogenotrophic and acetoclastic pathways. In summary, the core bacterial phyla in the thermophilic reactor exhibit a functional synergy essential for efficient anaerobic digestion. The hydrolytic and acidogenic activities of *Firmicutes* and *Thermotogota* provide a steady stream of simple substrates. *Cloacimonadota* facilitates the crucial syntrophic degradation of intermediate fatty acids, preventing process inhibition, while *Halobacterota* offers an additional pathway for methane generation. The coordinated metabolic activities of these phyla ensure a stable supply of substrates for the methanogenic community, thereby underpinning the stability and productivity of the entire TAD process.

**Fig 5:**
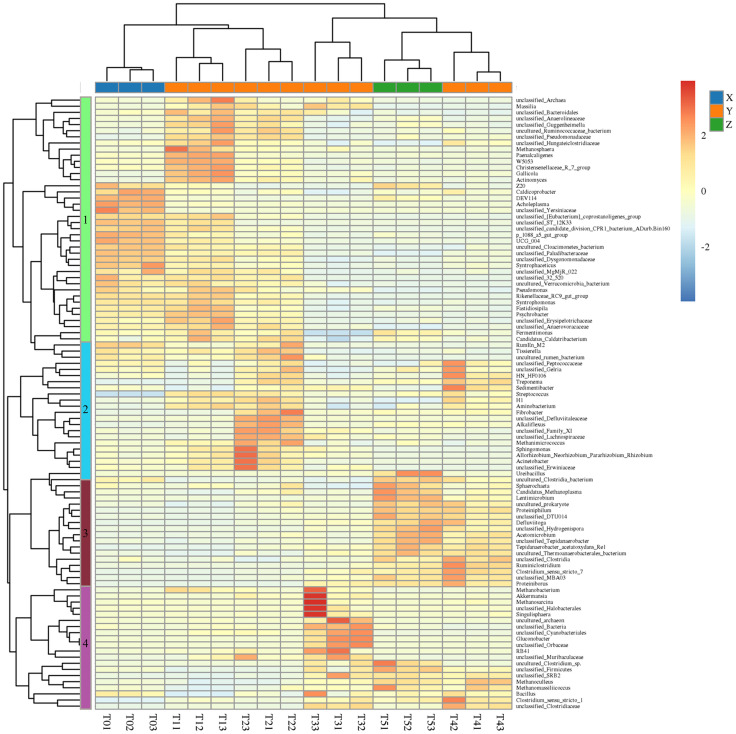
Heatmap analysis of microbial abundance changes at species level during temperature increase. Clustering of different microbial community was performed and each cluster is represented by a different color.

Fig 4b shows the differences in the mean proportions of the top 10 bacteria at the genus level. The average relative abundances of *Defluviitoga*, *Proteiniphilum*, *Methanoculleus* and *Methanoplasma* were significantly higher in T2-T5. *Defluviitoga* exhibited a significant increase in relative abundance during thermal ramp-up, with a 5.5-fold higher abundance in T5 compared to T2 (initial stabilization at 50 °C). *Defluviitoga* (Thermotogota), a polysaccharide-degrading bacterium, is a dominant species in TAD [34]. It hydrolyzes diverse carbohydrates into acetate, H₂, and CO₂, which methanogenic archaea then utilize to produce methane. Similarly, the high proportion of *Proteiniphilum* in T5 participated in the acidification stage for VFAs production and aggravated the accumulation of VFAs. As previously reported, *Proteiniphilum* (a kind of proteolytic bacteria) utilized proteins and carbohydrates for anaerobic microbial growth, linking proteolysis to downstream methane production [35]. Collectively, thermal stimulation enriched polysaccharide hydrolytic and proteolytic bacteria to promote digestion efficiency and maintain system stability. The accelerated hydrolysis process promoted VFAs accumulation, providing abundant substrates for methanogens. As shown in Fig 4b, *Methanoculleus* (17.1–26.9% relative abundance) emerged as the dominant genus in the thermophilic microbial community. Concurrently, *Methanoplasma* abundance increased significantly to 6.9% at 60 °C compared to the initial 2.8% at 50 °C. Heatmaps were employed for further functional analysis. The differential patterns of microbial community structure during the thermal ramp-up process were classified into four characteristic clusters (Clusters 1–4), each showing distinct microbiota abundance profiles (Fig 5). Overall, the inoculum and mesophilic fermentation systems shared highly similar microbial community structures (Cluster 1). Subsequently, the temperature surge to 50 °C (initiating thermophilic conditions) triggered microbial community reorganization, defining Cluster 2. The abundance of *Fibrobacter and Defluvitaleaceae* increased sharply, enhancing the hydrolysis of macromolecules (e.g., cellulose) and their subsequent conversion to acetate, a key volatile fatty acid (VFA). Correspondingly, the abundance of the methanogenic archaeon *Methanimicrococcus* increased synergistically to facilitate substrate conversion and enhance methane synthesis. After 10 days of stable thermophilic operation followed by gradual heating to 52 °C, thermophilic methanogens progressively enriched, establishing a stable high-temperature biogas-producing community (Cluster 4). *Mehanobacterium* (hydrogenotrophic methanogens), *Methanosarcina* (acetoclastic methanogens), Methanoculles (hydrogenotrophic methanogens) and *Methanomassiliicoccus* (methyl-reducing methanogens) were significantly enriched, which were closely correlated with enhanced methane yield (day10 ~ 20). Moreover, hydrogenotrophic methanogens dominated in relative abundance, consistent with prior studies demonstrating that the hydrogenotrophic methanogenic pathway is thermodynamically more advantageous than the acetoclastic pathway [36]. Furthermore, the hydrogenotrophic methanogens are more tolerant to changes in the digestive environment and the accumulation of ammonia and VFAs [37]. However, further temperature increases selectively enriched hydrolytic acidogenic bacteria (Cluster 3), such as *Clostridia bacterium, Sphaerochaeta, Lentimicrobium, Proteiniphilum, Defluvitoga,* and *Acetatoxydans*, whose metabolic activity progressively acidified the system. In AD systems, *Clostridia bacterium* dominates the acidogenic phase, significantly enhancing VFAs yields. Similarly, *Lentimicrobium* proliferation improves hydrolysis-acidification efficiency and stimulates ethanol and acetate production [38]. Under thermophilic conditions, *Defluvitoga, Sphaerochaeta* and *Acetatoxydans* demonstrate robust metabolic activity in high-efficiency AD reactors, further contributing to acetate generation [39–40].

### Correlation analysis between microbial community and environmental factors

**Fig 6:**
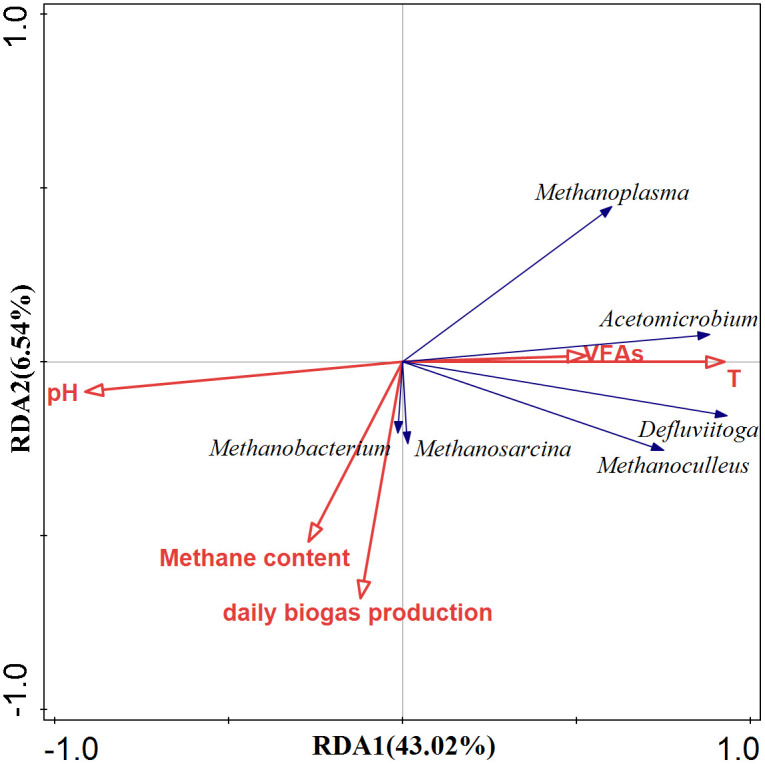
RDA of microbial communities and environmental factors throughout thermal transition. Microorganisms were selected at the genus level of each group. The black lines and red lines represent different bacteria and environmental factors, respectively.

To gain deeper insights into the impact of temperature on microbial communities and daily biogas production, redundancy analysis (RDA) was conducted (Fig 6). Temperature had a strong positive correlation with VFAs and increasing temperature promoted the abundance of hydrolysis-acidification bacteria *Defluvitoga* and *Acetomicrobium*. However, the daily biogas production and methane content showed negative correlations with both temperature and VFAs, suggesting that thermophilic AD experienced VFA inhibition. The daily biogas production and methane content had strong correlations with *Mehanobacterium* (hydrogenotrophic methanogen) and *Methanosarcina* (acetotrophic methanogens). These results indicated that the synergistic coupling of hydrogenotrophic and acetoclastic methanogenesis pathways significantly improved methane yield. Interestingly, temperature had almost no correlation with those two methanogens, but a positive correlation with *Methanoplasma* and *Methanoculleus*, mainly hydrogenotrophic methanogens. The composition of methanogenic communities exhibited considerable variation in different temperature. As demonstrated in previous studies, hydrogenotrophic methanogens exhibit superior adaptation to thermophilic anaerobic digestion systems [41].

Regarding the microbial community, *Defluviitoga* exhibited significant positive correlations with nearly all methanogens, suggesting a potential synergistic relationship. *Defluviitoga*, a dominant hydrolytic bacterium in anaerobic digestion systems, can provide sufficient nutrient substrates (e.g., H₂, CO₂, and acetate) for methanogens. *Methanobacterium* and *Methanosarcina* can utilize H₂/CO₂ and acetate, respectively, to produce CH₄. Additionally, *Methanosarcina* helps alleviate VFA accumulation, thereby enhancing daily biogas production. However, under thermophilic conditions, high enrichment of *Defluviitoga* combined with increased proportions of hydrogenotrophic methanogens collectively accelerated the acidification process in the fermentation system. Consequently, within a defined range, temperature increase helps maintain microbial community stability and sustains methane production. However, beyond a critical threshold, it induces significant structural shifts in the community, ultimately reducing methane yields.

### Performance of Thermophilic Reactors Under Different OLR

**Fig 7:**
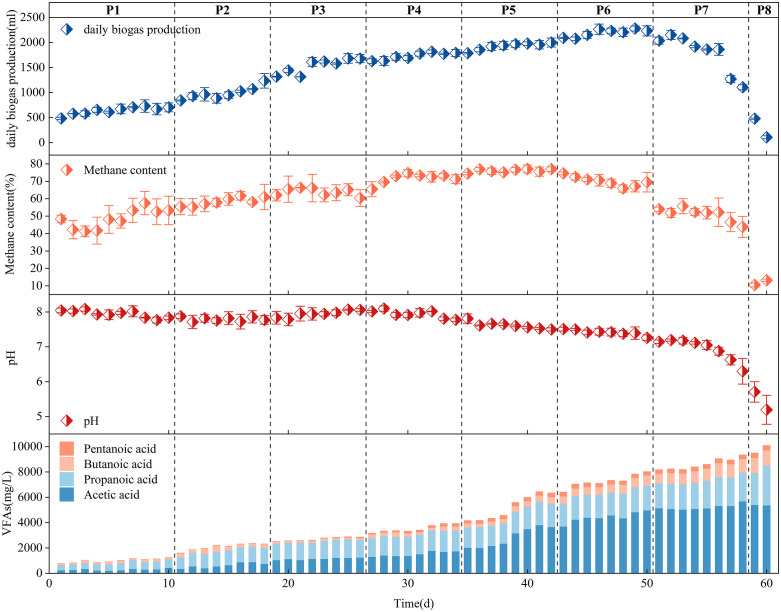
Thermophilic anaerobic digest performance under different OLRs. (a) daily biogas production; (b) methane content; (c) pH and; (d) VFA concentrations under different OLRs.

Based on comprehensive evaluation of both biogas production performance and microbial community dynamics, the thermophilic anaerobic digestion system was maintained at 52–55 °C for optimal operation. To further enhance methane production, thermophilic wet anaerobic digestion was conducted with varying OLRs. The initial OLR was set at 2 g VS/(L·d), with subsequent increases of 0.5 g VS/(L·d) per 8-day. The daily biogas production initially exhibited a significant upward trend from P1 to P3 (ramp-up phase) (Fig 7a), then stabilized during P4-P5 (stable phase). The peak daily biogas yield (2264.8 ml) was observed in P6, with the phase average (2,191.5 ml) representing a 3.43-fold increase over P1 (638.1 ml). However, when the OLR was increased to 4.5 g VS/(L·d), biogas production plateaued and then began to largely decline from the 57th day. Further elevation OLR to 5 g VS/(L·d) induced termination of anaerobic biogas production. These results align with Zhang et al.‘s findings, where the maximum OLR tolerance was 4 g VS/(L·d) in a thermophilic dry anaerobic co-digestion system [8]. Notably, variations in food waste source and composition led to significant differences in AD performance, particularly in biogas yield.

Methane content is another critical parameter in anaerobic digestion and the concentration of CH_4_ is generally 50%–70% [42]. The CH_4_ concentration remained stable at approximately 72−76% during P4 ~ P5 (stable phase), but exhibited an initial decline in P6 (critical unstable phase), despite the ongoing continuing in biogas yield during this period (Fig 7b). This was probably because OLR elevation enhanced the progressive accumulation of hydrolysis-derived intermediates (acetate, H₂, and CO₂), which exceeded the metabolic capacity of methanogens, ultimately causing the observed decline in CH₄ content. While in P7-P8 (unstable phase), the methane content declined to the minimum along with a reduction in biogas production. Throughout operation, pH remained stable within the optimal methanogenic range (6.8–8.0) except when OLR exceeded 4.5 g VS/(L·d) (Fig 7c). Elevated OLR levels led to incomplete VFA degradation, consequently reducing CH₄ production yield and final concentration. Increasing the OLR from 1.5 to 3.5 g VS/(L·d), TVFA concentrations were stable at 2.1–5.2 g/L, VFAs were fully degraded, and CH_4_ yields gradually increased. Meanwhile, the proportion of acetate ranged from 29.7% to 53.8%, providing aceticlastic methanogens with directly available substrates and increasing methane production. However, as the OLR increased to 4–4.5 g VS/(L ⋅ d), TVFA accumulation further, reaching 9.0 g/L by day 56, even though the pH did not change dramatically. As previous studies have reported, thermophilic AD systems maintain stability when VFA concentrations was below 10 g/L [43]. During the nonequilibrium phase P8, the significant accumulation of acetic acid (5.4 g/L), propanoic acid (2.5 g/L) and butanoic acid (1.2 g/L) occurred, triggering a sharp decrease in pH and ultimately causing the failure of thermophilic AD.

**Fig 8:**
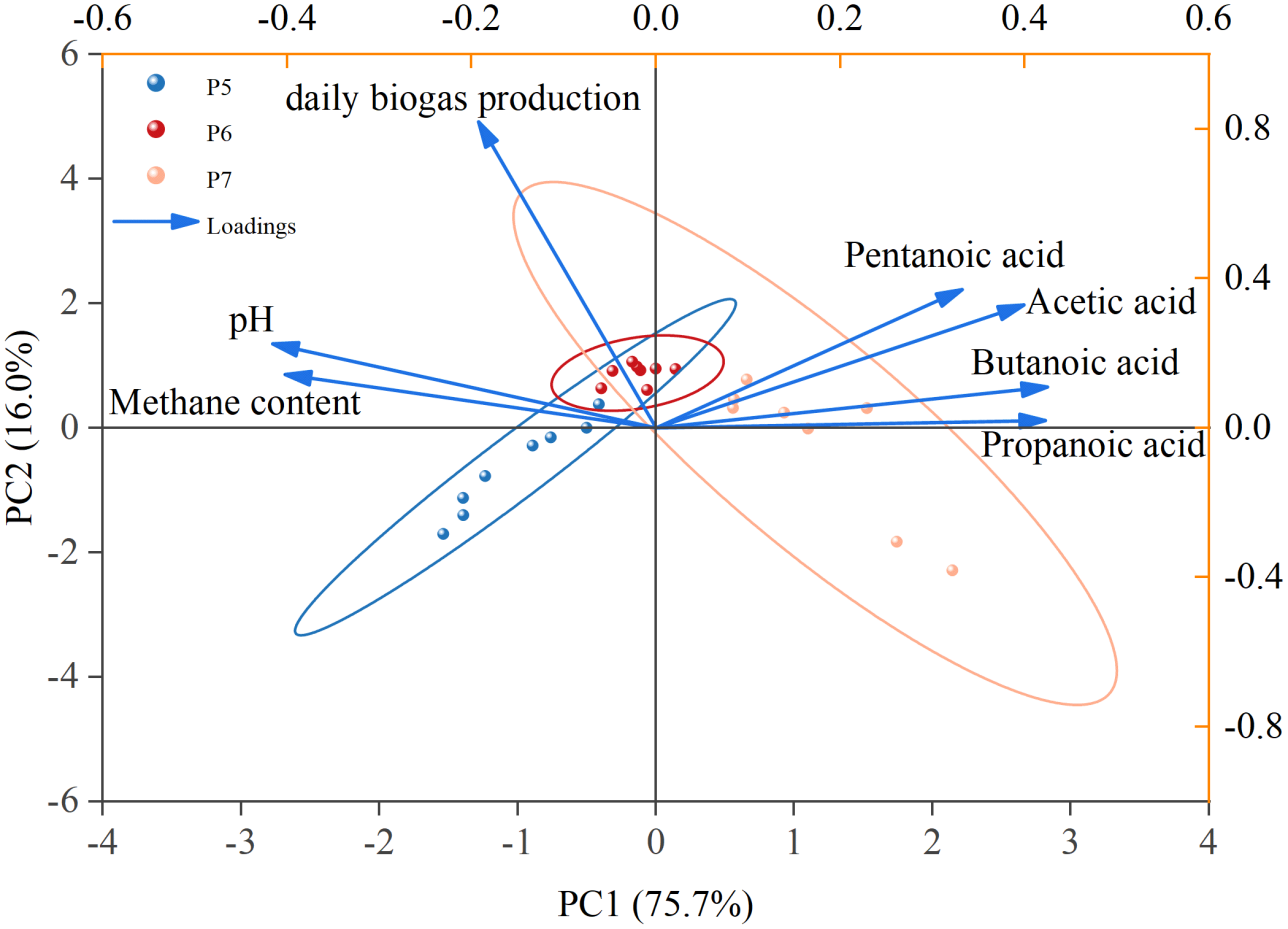
Principal Component Analysis of Three Typical Stages During Organic Loading Increase.

Principal component analysis (PCA) was performed on methane production and AD process parameters during the P5, P6, and P7 phases (representing three distinct stages: stable, critically unstable, and unstable) to reveal their correlations (Fig 8). Different fermentation phases were color-coded for distinction, while parameter relationships were indicated by loading vectors. The two principal components collectively explained 91.7% of the total variance. Principal component 1 (PC1), accounting for 75.7% of the variance, represented the dominant component and showed strong correlations with acetic acid, butyric acid, and propionic acid concentrations. Principal component 2 (PC2) was primarily associated with daily biogas production. Sample similarity was determined by inter-point distances or group ellipses. The sample groups showed cross-distribution patterns. In particular, the transitional P6 phase displayed significant ellipse overlap with both P5 and P7 phases, but only minimal overlap between the P6 and P7 ellipses. This distribution pattern was consistent with the variation trends in Fig 7. Moreover, the correlation between the process parameters shown biogas daily yield and methane content were significantly negatively correlated with butyric acid and propionic acid, but had the positive correlation with pH value. Given the sustained high hydrolysis rate in thermophilic AD, the mismatch of hydrolytic efficiency led to irreversible acidification, critically destabilizing the fermentation system. As previous studies demonstrated that pH fluctuations more significantly inhibit methanogenic activity than bacterial metabolic functions, ultimately reducing biogas yield [44]. Furthermore, PAC results suggested that butyric and propionic acid concentrations could serve as potential monitoring parameters for evaluating thermophilic AD stability.

These findings have direct implications for developing effective operational strategies. The PCA highlights that VFA concentrations are more sensitive early-warning indicators of instability than pH, whose decline often lags. Therefore, an effective monitoring strategy should prioritize the real-time tracking of VFAs (e.g., butyric and propionic acid) to detect any drift from the stable P5 state towards the critical P6 transition. Upon detection of such a shift, a dynamic control strategy is crucial to prevent irreversible acidification (the P7 state). The primary corrective action is the dynamic adjustment of the OLR: reducing or temporarily halting substrate feeding. This intervention allows the methanogenic community to consume excess VFAs, thereby restoring pH and guiding the system back to stability. Proactive alkalinity addition (e.g., with sodium bicarbonate) can serve as a valuable supplementary measure to enhance the system’s buffering capacity against transient stress events.

## Discussion

### A two-stage temperature-up-shift strategy for thermophilic microbiota enrichment

This study cultivated a high-performance thermophilic consortium from mesophilic inoculum via a two-step temperature-up-shift strategy for the anaerobic digestion of food waste. A mesophilic inoculum was selected for thermophilic adaptation based on its widespread availability from stable, full-scale digesters and the necessity to mitigate the high instability risks associated with direct thermophilic seeding. This gradual acclimatization approach, while initially slower, ultimately cultivates a robust consortium specifically tailored to the local substrate, ensuring greater long-term stability and superior performance compared to the high-risk, direct-seeding alternative. A two-stage temperature control strategy was employed to cultivate the thermophilic consortium. The initial ‘Rapid Screening’ phase involved an abrupt temperature up-shift from 37 °C to 50 °C. This gradual process facilitates the adaptation of slow-growing and environmentally sensitive methanogens, fostering a synergistic succession between acidogenic and methanogenic populations. The outcome is a robust microbial consortium that combines high degradation efficiency with stable methanogenic activity.

The acclimation strategy employed in this study successfully enriched a functional thermophilic consortium comprising key hydrolytic, acidogenic, and methanogenic guilds, confirming the efficacy of the approach. Consistent with its known role in thermophilic systems, the polysaccharide-degrading bacterium *Defluviitoga* (phylum Thermotogota) emerged as a dominant genus, with its abundance increasing significantly in response to thermal elevation. This enrichment was pivotal for the hydrolysis of complex carbohydrates into acetate, H₂, and CO₂—essential methanogenic precursors. Similarly, the high relative abundance of *Proteiniphilum* is consistent with its reported role in coupling the degradation of proteins and carbohydrates to downstream methanogenesis. Notably, the methanogenic community was dominated by the hydrogenotrophic archaeon *Methanoculleus* (Fig 4b), a pattern frequently observed in stable thermophilic digesters. Strikingly, however, our study revealed a significant enrichment of *Methanoplasma* at higher temperatures. This finding is particularly intriguing given that *Methanoplasma*, which utilizes methyl-compounds and H₂, is typically characterized as a mesophile ill-suited for thermophilic environments. We hypothesize that this unexpected enrichment was facilitated by the gradual, stepwise acclimation strategy, which may have provided an extended opportunity for this typically slow-growing archaeon to adapt. Furthermore, the ample availability of H₂ within the system, evidenced by transient accumulations observed during our monitoring, likely created a unique ecological niche. This niche selectively promoted the proliferation of a previously uncharacterized thermotolerant species or strain of *Methanoplasma*. This co-enrichment of *Methanoculleus* and *Methanoplasma* forged a robust and distinct hydrogenotrophic/ methylotrophic methanogenic partnership. The synergistic increase of these archaea in specific proportions with the hydrolytic and acidogenic bacteria established a highly specialized functional consortium. This consortium was uniquely adapted to the thermophilic (55 °C) digestion of food waste, culminating in enhanced methanogenesis performance.

The boundaries of this acquired thermotolerance were, however, defined by the system’s acidogenic collapse upon a temperature increase to 60 °C. Nevertheless, the system demonstrated notable resilience, with methanogenic activity resuming within three weeks of reverting to 55 °C and ceasing substrate loading, indicating the restoration of the community’s functional capacity. This observation underscores that advancing the thermal limits of such systems necessitates an even more refined, gradual acclimation protocol. These results suggest that future success will depend on prolonging stabilization periods at each temperature increment and employing adaptive management strategies, such as reverting to previous stable conditions at the first sign of process instability (e.g., VFA accumulation). Ultimately, achieving a robust and resilient thermophilic consortium requires not only reaching a target temperature but also maintaining prolonged operation to ensure the community’s long-term stability and performance.

### Operation and performance of thermophilic anaerobic digestion of food waste

Consistent with the established literature, this study confirms that TAD enhances microbial metabolism, accelerating the rate-limiting hydrolysis of complex organic matter and consequently leading to higher volumetric biogas yields. As a critical operational parameter in TAD, the OLR directly influences both process efficiency and stability. A stepwise increase in OLR can significantly enhance volumetric methane production, thereby maximizing the reactor’s treatment capacity. In the present study, following the establishment of a stable thermophilic consortium, the OLR was successfully increased stepwise from 1.5 to 4.0 g VS/(L·d). This strategy resulted in a stable, high-performance digestion system, achieving a peak daily biogas yield of 2264.8 mL and a high methane concentration of 72–76%. However, when the OLR was further increased to 4.5 g VS/(L·d), process inhibition occurred. The balance between acidogenesis and methanogenesis was disrupted, leading to the rapid accumulation of VFAs and a subsequent decline in pH, which ultimately caused a reduction in the system’s methanogenic activity and a decrease in methane production.

While this study defines a safe and stable operational OLR for a TAD system, this threshold can be significantly extended through strategic interventions, including the optimization of feedstock composition, microbial acclimation, and nutrient supplementation. One of the most effective strategies is co-digestion demonstrated that while their mono-digestion of food waste failed at an OLR above 3 g VS/(L·d), co-digesting the same waste with kitchen or garden waste successfully increased the OLR tolerance to 4 g VS/(L·d) [12]. The co-substrates likely mitigated the accumulation of inhibitors, such as ammonia and VFAs, and provided a more balanced nutrient profile. This highlights how feedstock synergy can fundamentally enhance system resilience and productivity. Another critical pathway involves long-term adaptation combined with nutrient supplementation. Stable TAD of food waste has been achieved at an exceptionally high OLR of 10.0 kg-COD/(m³·d) through a prolonged 230-day operation with a stepwise OLR increase and, crucially, the supplementation of trace elements [45]. Furthermore, a comparative study found that as the OLR increased from 2.5 to 5.5 g VS/(L·d), a thermophilic reactor outperformed its mesophilic counterpart, but clear process inhibition occurred at 6.5 g VS/(L·d) [10]. Therefore, future efforts to optimize high-rate TAD of food waste should focus on integrated strategies, including improving substrate characteristics through co-digestion, selecting for robust microbial consortia via long-term acclimation, and supplementing essential micronutrients to support methanogenic activity under high-load conditions.

However, maximizing the OLR addresses only one aspect of process viability. The ultimate feasibility of any high-rate TAD system hinges on its thermodynamic performance—specifically, whether it can achieve a positive net energy balance. While the TAD process offers intrinsic benefits, it simultaneously presents a thermodynamic paradox where the high energy output from methane is offset by the substantial energy expenditure needed to sustain elevated temperatures. To this end, an energy balance analysis of our laboratory-scale process at its peak stable state (55 °C, 4 g VS/(L·d) OLR) was performed. The analysis revealed that the system generated 55.1 kJ/d (E_out) from methane production, whereas the energy input (E_in) was dominated by thermal losses (Q_loss) attributable to the reactor’s high surface-area-to-volume ratio. These losses far exceeded the modest 2.8 kJ/d required for influent heating (Q_feed), resulting in a negative net energy balance (E_net < 0). This outcome is a characteristic artifact of bench-scale systems where validating process performance, rather than achieving energy self-sufficiency, is the primary objective. This energetic limitation is overcome in large-scale industrial applications, where the exponentially lower surface-area-to-volume ratio enables significant heat retention and ensures a positive net energy yield.

## Conclusions

This study presents a robust stepwise heating strategy for effectively acclimating a widely available mesophilic inoculum for high-performance TAD, thereby overcoming the common challenges of inoculum scarcity and startup instability. The optimal operational temperature for methane production was determined to be 52–55 °C. This thermal condition promoted the enrichment of the dominant carbohydrate-hydrolytic bacterium *Defluviitoga* and established a stable methanogenic community dominated by hydrogenotrophic methanogens, which significantly enhanced methane productivity. Under these optimized conditions, a stepwise increase in the OLR from 1.5 to 4.0 g VS/(L·d) substantially improved performance, culminating in a peak daily biogas yield of 2264.8 mL with a stable CH₄ concentration of 72–76%. Process inhibition was observed when the OLR exceeded 4.5 g VS/(L·d), defining a critical operational threshold. Critically, PCA revealed that VFA concentrations—specifically butyric and propionic acid—are more sensitive early-warning indicators of system instability than pH. This finding offers a proactive monitoring strategy, enabling operators to adjust the OLR to prevent irreversible acidification and system collapse.

## Supporting information

S1 Fig Microbial beta-diversity patterns during the temperature-rising process. (PNG)

S2 Fig Organic matter removal and conversion rates during temperature increase in anaerobic fermentation of food waste. (TIF)

S1 Appendix List of alpha diversity indices during the temperature-increasing process. (XLS)

S2 Appendix Raw data for Replication of study results. (XLSX)
