# Supplementary figures and images for: Thermophilic microbiome acclimation for enhanced anaerobic digestion of food waste: Optimization and performance evaluation
Source: PLoS One. 2025 Nov 10;20(11):e0336355. doi: 10.1371/journal.pone.0336355 (PMC12599915; doi:10.1371/journal.pone.0336355)

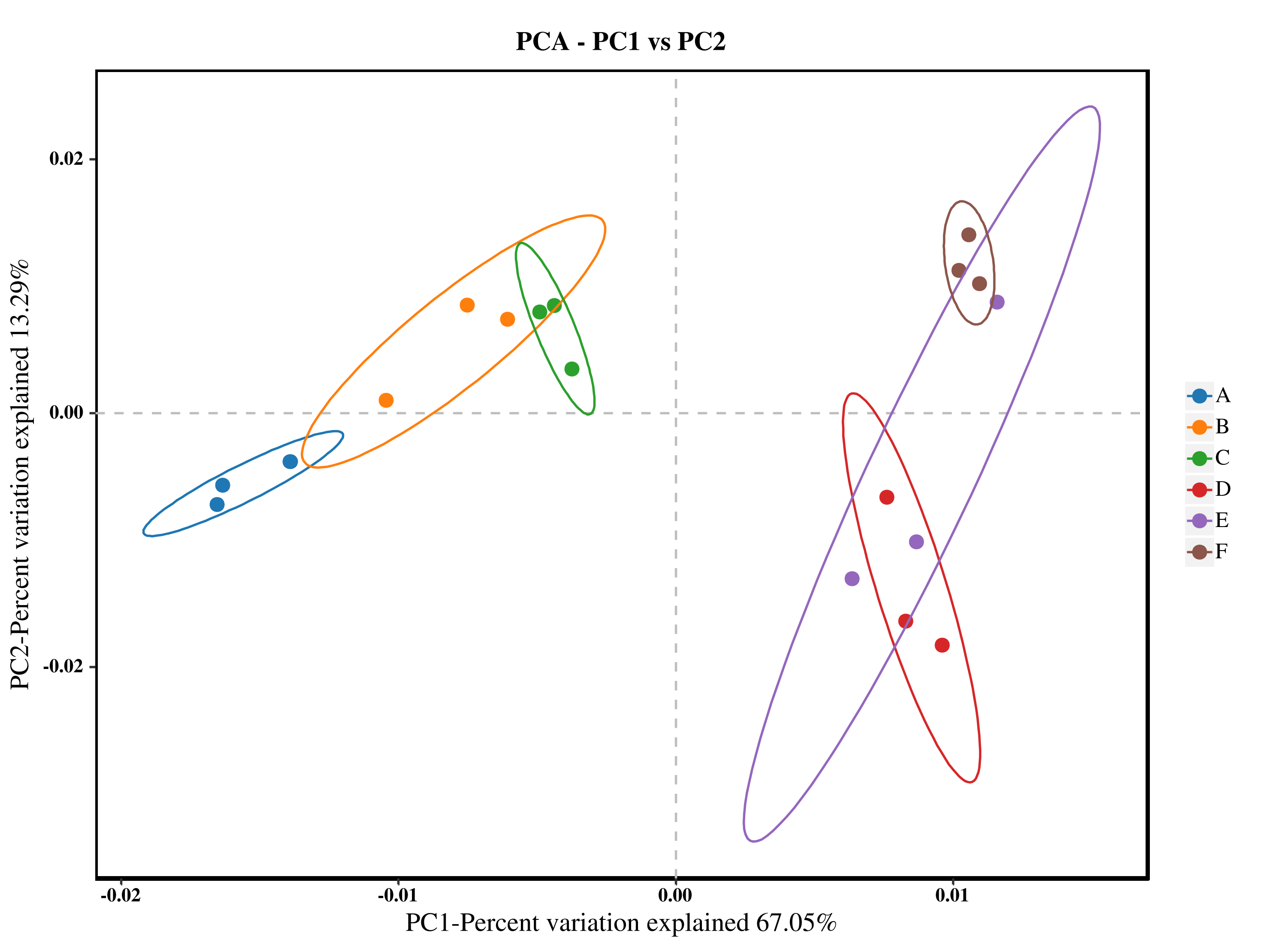

Supplement: S1 Fig — (PNG) [file pone.0336355.s001.png]
